# Identification of Linear B-Cell Epitopes on Hemagglutinin Protein of Canine Distemper Virus Using Two Monoclonal Antibodies

**DOI:** 10.3389/fvets.2020.00047

**Published:** 2020-02-28

**Authors:** Pengfei Shi, Zhigang Cao, Yuening Cheng, Shipeng Cheng, Li Yi

**Affiliations:** Institute of Special Economic Animal and Plant Science, Chinese Academy of Agricultural Sciences, Changchun, China

**Keywords:** canine distemper virus, hemagglutinin protein, monoclonal antibody, B-cell epitope, conservation analysis

## Abstract

Canine distemper virus (CDV) belongs to the *Morbillivirus* genus of the Paramyxoviridae family, which causes a threat to the domestic dog and fur-animal industry. Hemagglutinin protein is a major membrane protein of the vital molecular factor in CDV tropism, also known to induce hosts to produce neutralizing antibodies. In the current study, we prepared two monoclonal antibodies, 1A5 and 2B8, against the H protein of the CDV-PS strain. A series of partially overlapping synthetic peptides covering the hemagglutinin protein (amino acids 50–204) were screened to define the linear epitope identified by 1A5 and 2B8 mAbs. ^120^QKTNFFNPNREFDFR^134^ (F8) and ^178^ARGDIFPPY^186^ (F14-1) are minimal linear epitopes recognized by 1A5 and 2B8 mAbs, respectively. Further investigations revealed that F8 is conserved in different CDV strains; however, F14-1 contains mutant residues 178, 179, and 180. The epitopes F8 and F14-1 localized at the surface of hemagglutinin protein in a three-dimensional (3D) structure. CDV-infected dog serum can also recognize the identified B-cell epitopes.

## Introduction

Canine distemper is a contagious disease with multisystem infection caused by canine distemper virus (CDV), which affects the health of multiple species (1, 2). The disease is characterized by high fever, diarrhea, encephalitis, immunosuppression, and other symptoms. The high mortality and morbidity of dogs and fur animals result in serious economic losses. The emergence of canine distemper has been continuously reported in China (3). Therefore, controlling the spread of CDV still faces enormous challenges. CDV is an enveloped, non-segmented, single-stranded negative RNA virus with a genome size of ~15.7 kb. The genome encodes nucleocapsid protein (N), phosphoprotein (P), matrix protein (M), fusion protein (F), hemagglutinin protein (H), and large protein (L). The CDV envelope consists of two integral glycoproteins, fusion protein (F) and hemagglutinin protein (H) (4). The H gene contains 1,947 nucleotides, encoding 607 amino acids. The size of CDV H protein is ~78 kD. Embedded on the surface of the viral envelope, H protein can effectively induce the body to produce neutralizing antibodies and play an important role in antigenic recognition (5). Recently, many studies reported the application of H protein in vaccines and diagnosis (6, 7). The antigenic epitopes of CDV hemagglutinin protein have not been investigated clearly.

In this study, two B-cell epitopes in H protein were identified by using specific mAbs to react with a series of truncated peptides in ELISA test. The epitope F8 is highly conserved among different isolates of CDV, and the epitope F14-1 contains three mutant residues. The localization of two epitopes on the surface of H protein makes it easy for F8 and F14-1 to induce an immune response during CDV infection. Furthermore, the current work actually provides potential uses for the development of diagnostic methods to detect CDV.

## Method

### Cell Lines, Viruses, and Serum Samples

Vero cells were cultured in Dulbecco's Modified Eagle's Medium (DMEM, Corning), which were supplemented with 10% fetal bovine serum (FBS) (Gibco-BRL, USA), at 37°C under a humid 5% CO_2_ atmosphere. Two monoclonal antibodies, named 1A5 and 2B8, were previously constructed and stored in the lab. These two monoclonal antibodies were produced by immunizing mice with recombinant truncated CDV-PS strain H protein. The plasmid pEasy-H protein aa 1–204 was transformed into *Escherichia coli* BL21 (DE3) competent cells (Takara) for protein expression (Figure S1). According to standard procedures, truncated H protein (1–204 aa) was used as the antigen to immunize mice to produce CDV H–specific mAb (8). CDV-PS (GenBank accession number: JN896331.1), CDV-Rockborn (GenBank accession number: GU810819.1), and CDV3 (GenBank accession number: EU726268.1) were used for cell inoculation in immunofluorescence assay (IFA) and WB tests. To determine the virus titers, cells cultivated in 96-well plates were inoculated with 10-fold serial dilutions of the virus and incubated at 37°C for 3–5 days. The viral titers were estimated with the Reed and Muench method and expressed as the 50% tissue culture infective dose (TCID_50_)/ml. The multiplicity of infection (MOI = 0.1) was confirmed according to the virus titer of the Vero cells. Dog serum samples collected from pet hospitals were confirmed to be CDV positive or negative by using colloidal gold immunochromatography test strips.

### Western Blotting

The two mAbs were analyzed by western blotting with Vero cells that had been infected with CDV-PS, CDV-R, and CDV3. Approximately equivalent amounts of each protein were separated by 12% SDS-PAGE gels. And then the separated proteins were electrophoretically transferred to a PVDF membrane (Merck Millipore, Germany) with constant electricity. After blocking the membrane with 5% skim milk in TBST for 2 h at 37°C, the membranes were incubated with 1A5 and 2B8 (diluted 1:2,000 in 5% skim milk in TBST) at 37°C for 2 h, respectively. They were washed three times with TBST and then probed with a 1:2,000 dilution of horseradish peroxidase (HRP)–conjugated goat anti-mouse IgG (CWBIO, China) at 37°C for 1 h. The immunoreactivity was visualized using a DAB kit (CWBIO, China).

### Immunofluorescence Assay

For IFA, Vero cells were, respectively infected with CDV-PS, CDV-R, and CDV3 in a 24-microtiter plate. And uninfected cells were used as negative control. After 36 h, the cells were fixed with 4% paraformaldehyde and then permeabilized with 0.1% Triton X-100. After blocking with 1% bovine serum albumin (BSA), the cells were probed with mAbs 1A5 and 2B8 for 2 h. After washing three times with PBST, the cells were incubated with fluorescein isothiocyanate (FITC)–conjugated goat anti-mouse IgG (Abclonal, China). Finally, the plate was viewed by a fluorescence microscope (Nikon TS100, Japan).

### Indirect ELISA

The mAb titers of 1A5 and 2B8 were determined by the established indirect ELISA method. Ninety-six-well plates were coated with CDV-PS (MOI = 0.1) 100 μl at 4°C and blocked for 2 h with 5% skim milk in PBS at 37°C followed by washing three times with PBST (containing 0.05% Tween 20). Then 96-well plates were inoculated with 2-fold PBS dilutions (the first well with 200-fold dilutions) of the mAbs and incubated at 37°C 1 h. After the incubation, 100 μl of HRP-conjugated goat anti-mouse IgG was added at a dilution of 1:2,000, and the sample was incubated at 37°C for 1 h. The reaction was quantified by measuring the absorbance at 450 nm using tetramethylbenzidine (TMB) as a substrate. Plates were coated with the mAb against canine parvovirus as the negative control. A value of P/N > 3 (positive/negative OD_450_ value) was the standard for the titer of mAbs.

### Identification of the Epitope's Position on Protein H Sequence

Through identification of the linear epitope with indirect ELISA, 15 overlapping synthetic peptides (15 aa) corresponding to the coding sequence of the truncated H protein (amino acids 50–204) were synthesized. Each 15 aa peptide overlapped the previous peptide sequence by 5 aa residues. An indirect ELISA was used to scan peptides 1–15. Ninety-six-well plates were coated with 1.0 μg/ml of each synthesized peptide at 4°C overnight. Each coated plate was blocked with 1% BSA in PBS for 2 h at 37°C. After blocking, aliquots of 100 μl mAbs (1:1,000 dilutions in PBST) were incubated at 37°C for 1 h. HRP-conjugated goat anti-mouse IgG (100 μl) was added at a dilution of 1:2,000, and the sample was incubated at 37°C for 1 h. The reaction was quantified by measuring the absorbance at 450 nm using TMB as a substrate. To further determine the minimal epitopes, we developed progressive single amino acid deletion from the N- or C-terminus of the result of this initial screen. The more refined mapping of the minimal linear peptide epitopes recognized by the mAbs using the ELISA method is described above.

### Biological Information Analysis

To investigate the homology of the epitope to CDV sequences, sequences corresponding to the region encompassing the mAbs' truncated B-cell epitopes were aligned with other representative strains which were available in the NCBI protein database (https://www.ncbi.nlm.nih.gov/protein/). The 17 CDV strains selected randomly contain different types, such as Asia-1, Asia-2, Vaccine, Europe, America-2, and Arctic. The nucleotide sequences of 24 CDV strains of H gene were available from GenBank, and a phylogenetic tree was constructed using the MEGA 7 software neighbor-joining method. GenBank accession numbers and genotypes of all isolates used in this study are listed in Table S1. The three-dimensional (3D) structure of identified epitopes in H protein was analyzed by mapping epitope locations onto a 3D model using PyMOL software based on the results obtained from the SWISS-MODEL online server.

### Reactivity of the Two Epitopes With Dog Serum Samples

To assess whether the epitope is immunodominant in serum, the sera were analyzed with indirect ELISA coated with ^178^ARGDIFPPY^186^ (F14-1) and ^120^QKTNFFNPNREFDFR^134^ (F8). And peptide F14-5 was the negative control. Ninety-six-well plates were coated with 100 μl ^178^ARGDIFPPY^186^ and ^120^QKTNFFNPNREFDFR^134^ (1.0 μg/ml) at 4°C overnight, respectively. Each coated well was blocked with 1% BSA in PBS for 2 h at 37°C. Positive serum and negative serum (100 μl) were combined with each well for 1 h at 37°C. After the incubation, 100 μl of HRP-conjugated rabbit anti-dog IgG was added at a dilution of 1:2,000, and the sample was incubated at 37°C for 1 h. TMB was used as a substrate for HRP. The absorbance was measured at 450 nm using the hybrid reader.

### Statistical Analysis

A Student *t*-test was used to analyze significant differences of OD value between positive and negative serum samples, or between control and mAbs 1A5 and 2B8 in indirect ELISA. Differences were analyzed by GraphPad Prism software (version 7.0) and were considered statistically significant at a *P* < 0.05.

## Results

### Identification and Characterization of mAbs Against CDV H Protein

**Figure 1:**
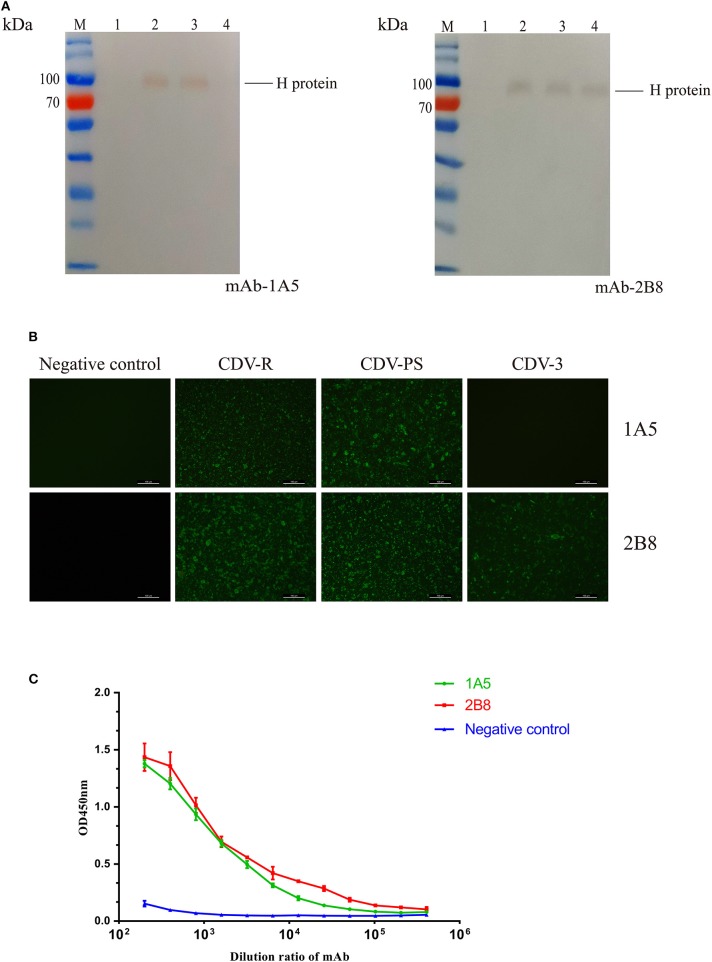
Characterization and identification of the mAbs. **(A–C)** The immunoreactivity of native canine distemper virus (CDV) H protein to the mAbs was analyzed with western blotting. **(A)** Lane M: PageRuler^TM^ Prestained Protein Ladder (Thermo Scientific); lane 1, cell lysates of uninfected Vero cells; lane 2, cell lysates of CDV-Rockborn–infected Vero cells; lane 3, cell lysates of CDV-PS–infected Vero cells; lane 4, cell lysates of CDV3-infected Vero cells. The mAbs are labeled at the right under each panel. **(B)** The mAbs recognized CDV-infected Vero cells by immunofluorescence assay (IFA). The cells were uninfected and infected with CDV. After 36 h, the cells were primed with 1A5 and 2B8. **(C)** Binding affinities of the mAb 1A5 and mAb 2B8 determined by indirect ELISA with CDV-PS strain. The value of OD_450_ nm is shown as a function of the respective dilution ratios of mAbs. The negative control used mAb against canine parvovirus.

The western blotting results showed that mAb 2B8 reacted with CDV-PS, CDV-R, and CDV3, while the 1A5 only recognized CDV-PS and CDV-R. Both of the mAbs did not recognize uninfected Vero proteins (Figure 1A). And the approximate size of the CDV H protein recognized by them is the same as the expected 78 kD. IFA demonstrated that 1A5 reacted with Vero cells infected with CDV-PS and CDV-R (Figure 1B). The mAb 2B8 reacted with CDV-PS, CDV-R, and CDV3. And neither of them reacted with uninfected Vero cells. These results indicate that two mAbs can specifically recognize CDV H protein. The mAb titer of 1A5 and 2B8 was determined by indirect ELISA method. The results showed that the titers were 10^4^ and 10^5^ (Figure 1C). These results indicate that mAbs 1A5 and 2B8 have good specificity and sensitivity for the development of diagnostic methods to detect CDV.

### Identification of B-Cell Epitopes Recognized by the Two CDV H Protein mAbs

**Figure 2:**
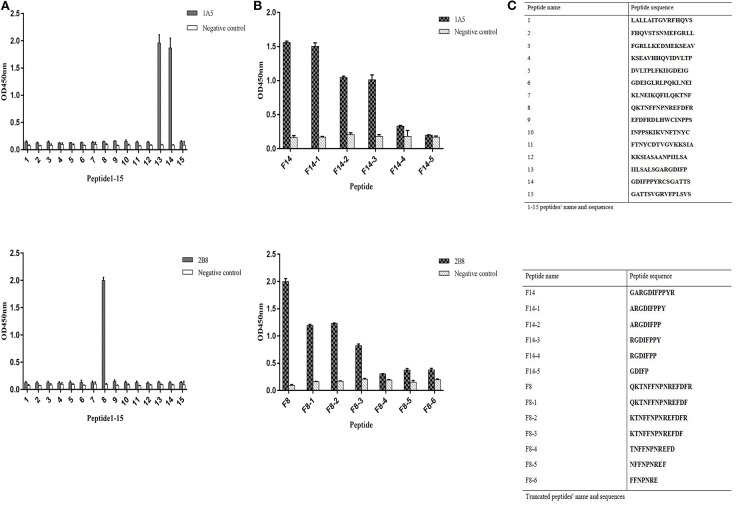
Identification of the epitope recognized by hemagglutinin (H)-reactive mAbs. **(A)** H protein–reactive mAbs were screened by indirect ELISA against a panel of 15 overlapping peptides derived from the CDV H protein (aa 50–204). The mAbs are listed in the upper right-hand corner of each bar graph. The Pc11 mAb was used as a negative control. The error bars display the standard deviation of three experimental repeats (*n* = 3). **(B)** The mAbs were screened against a series of progressively truncated peptides based on the results of the initial screen shown in the panel. The mAbs are labeled on the top right corner of each bar graph. The Pc11 mAb was used as a negative control antibody. The error bars display the standard deviation of three experimental repeats (*n* = 3). **(C)** The name and sequence of the peptides used are included in the table.

In Figure 2A, mAb 1A5 recognized the sequence ^170^IILSALSGARGDIFP^184^ (peptide13) and ^180^GDIFPPYRCSGATTS^194^ (peptide14). The core linear epitope might be represented by the sequence ^180^GDIFP^184^, which was the overlapping H protein sequence present in both peptides. The location of epitopes recognized by 2B8 is between amino acids 120 and 134 of H protein (Figure 2A). To further determine the minimal epitopes, we developed progressive single amino acid deletion at the N- or C-terminus of the ^177^GARGDIFPPYR^187^ (F14) and ^120^QKTNFFNPNREFDFR^134^ (F8) peptide sequence. A significant decrease could be observed when the peptide changed from F14-1 to F14-2. In Figure 2B, the OD_450_ value decreased from 2.00 to 1.19 when there was resection from the amino terminus of the polypeptide ^120^QKTNFFNPNREFDFR^134^. The minimum linear epitope recognized by 1A5 was ^178^ARGDIFPPY^186^, and it was ^120^QKTNFFNPNREFDFR^134^ for 2B8. The names and sequences of the peptides used are included in Figure 2C.

### Alignment and Conservation Analysis of the Defined Epitope

**Figure 3:**
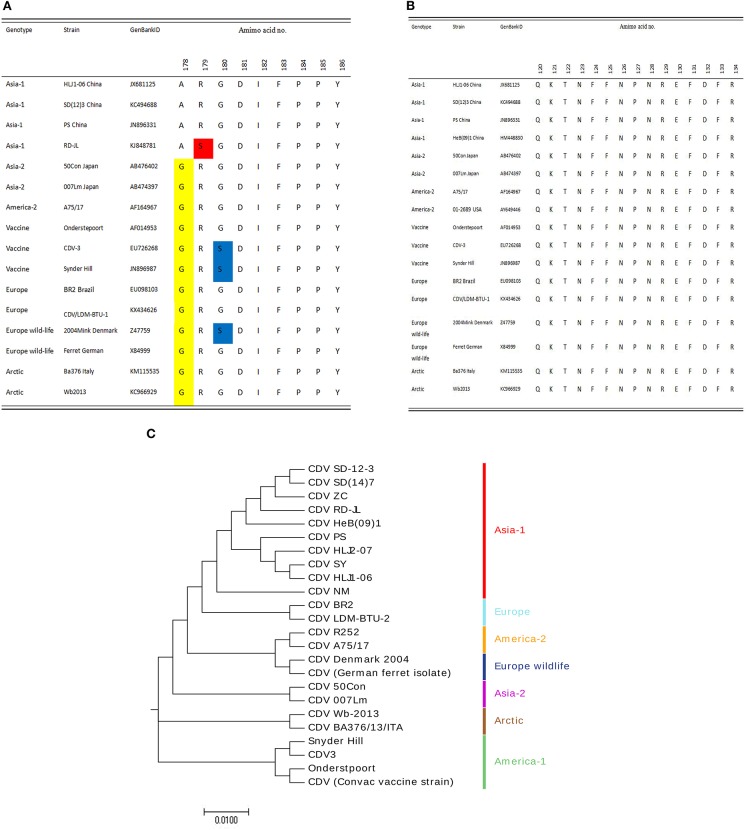
Conservation analysis of the epitopes. **(A)** Conservation analysis of the epitope F14-1 among different CDV strains. The yellow highlight indicates the difference on 178 amino acid identity, the red highlight shows the difference on 179 amino acid identity, and the mutation on 180 amino acids with blue highlight. **(B)** Conservation analysis of the epitope F8 among different CDV strains. **(C)** Genotyping of 24 CDV strains based on H protein sequences. The phylogenetic tree was constructed using the neighbor-joining method in MEGA 7.0 with bootstrap values from 1,000 resamplings shown for each node. GenBank accession numbers of all isolates used are listed in Table S1.

The B-cell epitope recognized by 1A5 is not conserved among CDV strains (Figure 3A); however, the minimal linear epitope by 2B8 is highly conserved among these representative CDV isolates (Figure 3B). To the epitope F14-1, the mutation occurs at 178 (yellow), 179 (red), and 180 (blue) amino acids. Combining phylogenetic tree (Figure 3C) and sequence alignment analysis, the epitope F14-1 is conserved in most Asia-1 genotype CDV strains except the CDV RD-JL strain. And the sequence alignment from 24 CDV strains revealed that the linear B-cell epitope of the mAb 2B8 is highly conserved among different CDV strains.

### Spatial Location of the Epitopes

**Figure 4:**
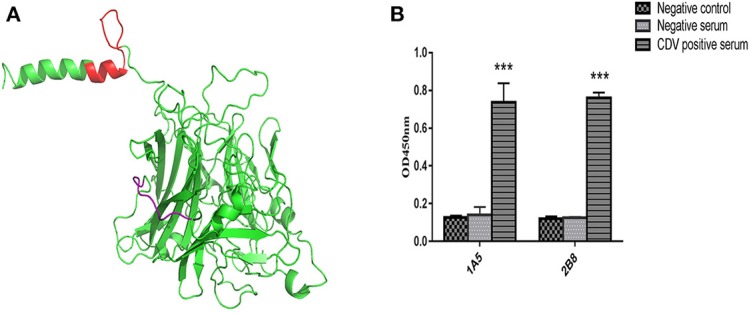
**(A)** Structural location analysis of epitopes on the CDV H protein (obtained from the Protein Data Bank). Location analysis of the identified B-cell epitopes using PyMOL software. The epitope ^178^ARGDIFPPY^186^ is labeled and shown in purple, and the ^120^QKTNFFNPNREFDFR^134^ location is shown in red. **(B)** Serological test for CDV H protein and the identified epitopes. The ^178^ARGDIFPPY^186^ epitope specific antibody and the ^120^QKTNFFNPNREFDFR^134^ epitope in anti-CDV sera were detected by peptide-coated indirect ELISA (*n* = 3). Asterisk indicates significant differences between CDV-positive sera and CDV-negative sera, or between controls (*P < 0.05; **P < 0.01; ***P < 0.001).

Modeling of the CDV H protein was conducted to spatially localize the epitopes recognized by mAbs 1A5 and 2B8. The 3D structure of the head and stalk region of the H protein is not available. However, we were able to localize the identified epitopes on a predicted 3D structure (SWISS-MODEL online server). The structural model demonstrated that the epitopes recognized by mAbs are shown to be fully exposed on the surface of the protein (Figure 4A). ^120^QKTNFFNPNREFDFR^134^ (red) is near the start region of the N-terminal domain. The structural results imply that the epitope sequences F14-1 and F8 are meaningful B-cell epitopes on the H protein of CDV.

### Reactivity of the Epitopes With Dog Serum

The minimal identified B-cell epitopes can be recognized by positive dog serum. Conversely, the OD_450_ value of negative serum was reduced significantly (*P* < 0.001). The same trend also showed in negative control peptides of H protein. According to the data of Figure 4B, we can draw the conclusion that identified B-cell epitopes are targeted to dog immune response during the virus infection.

## Discussion

Since the first isolation of CDV by Carré in 1905, CDV still remains a challenge to veterinarians and pet owners (9). Hemagglutinin protein is the suitable target to study genetic divergence and molecular epidemiology due to high mutation of the H gene (10, 11). H protein also plays an important role in interaction with host proteins like SLAM or nectin-4 (12). A series of studies have performed linear B-cell epitope mapping of CDV using monoclonal antibodies against N and P proteins (13, 14). And the antigenic differences between vaccine strains and wild-type strains have been reported. Due to antigenic differences between the vaccine strains and the currently circulating wild-type strains, current vaccines may provide incomplete protection (15). So it's necessary to clarify the detailed sequence of linear B-cell epitopes of the CDV H protein.

In this study, we generated two mAbs against CDV H protein and identified the characteristics of mAbs 1A5 and 2B8 by WB (Figure 1A) and IFA (Figure 1B). Furthermore, mAb 1A5 specifically binds epitopes with the exact core unit F14-1, and mAb 2B8 recognizes F8. Here the epitope ^120^QKTNFFNPNREFDFR^134^ was found to be conserved in different genotypes of CDV (Figure 3B). The IFA and WB test confirmed the sequence alignment results. Sequence alignment and phylogenetic analysis showed that ^178^ARGDIFPPY^186^ was conserved among most Asian-1 CDVs except CDV RD-JL (Figure 3A). So the specific mAb 1A5 can be used as a tool to differentiate CDV RD-JL from other CDV Asia-1 strains. The results of western blotting and conservation analysis of the epitopes demonstrated that the monoclonal antibody lost its characteristics when Ala became Gly at 178 and Gly became Ser at 180. The residues 178 and 180 may be the key determinants of F14-1 reactivity. Previous research indicated that the length of an epitope is usually 5 to 7 amino acids or does not exceed 20 amino acids (16). In this study, the minimal linearized epitopes were 9 and 15 amino acids, which are consistent with the results obtained in the previous study. In addition, we used peptides of identified epitopes to verify the reactivity of CDV-positive dog serum. The result demonstrated that CDV-positive dog serum was able to recognize the identified B-cell epitopes.

In summary, we generated two novel monoclonal antibodies (1A5, 2B8) and identified a highly conserved linear B-cell epitope against CDV H protein. The defined epitopes will enrich understanding of the immunological epitopes present in the CDV H protein. The study may also be useful for further developing the diagnostic tools for CDV infections.

## Data Availability Statement

All datasets generated for this study are included in the article/Supplementary Material.

## Ethics Statement

The animal study was reviewed and approved by Review Board Institute of Special Animal and Plant Sciences, Chinese Academy of Agricultural Sciences (CAAS).

## Author Contributions

PS and LY designed the study. PS and ZC performed the experiments. YC and SC analyzed and prepared the figures and tables. PS wrote the manuscript.

### Conflict of Interest

The authors declare that the research was conducted in the absence of any commercial or financial relationships that could be construed as a potential conflict of interest.
