# Supplementary material for: Identification of Linear B-Cell Epitopes on Hemagglutinin Protein of Canine Distemper Virus Using Two Monoclonal Antibodies
Source: Front Vet Sci. 2020 Feb 28;7:47. doi: 10.3389/fvets.2020.00047 (PMC7058631; doi:10.3389/fvets.2020.00047)
Supplement: Supplementary file 1 [file Data_Sheet_1.pdf]

Supplementary Material

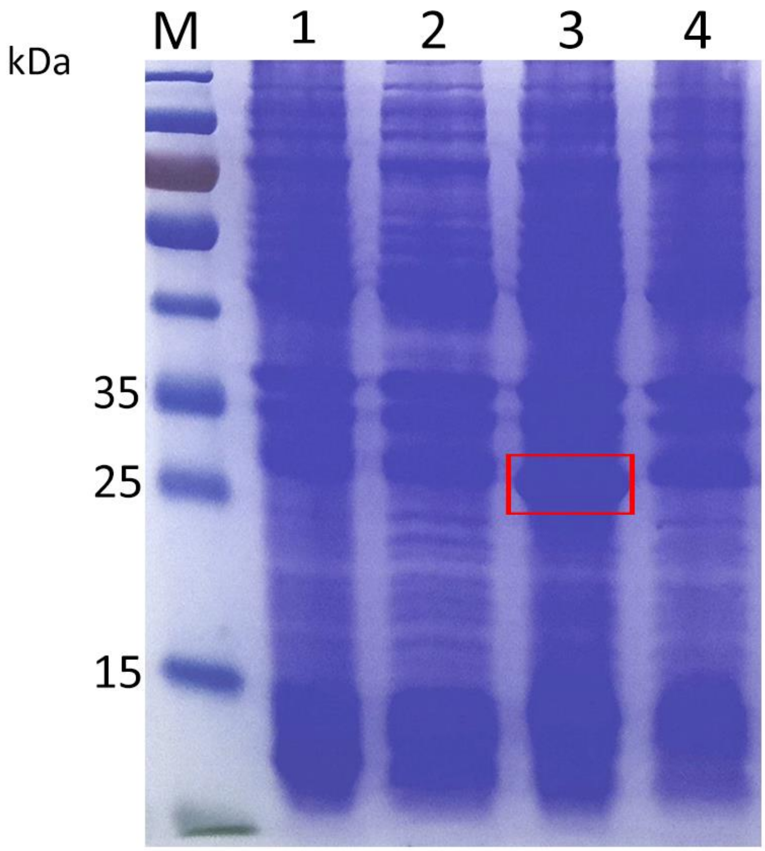

FIGURE 1 Expression of His-tagged H protein 1-204aa. Bacterial lysates from E. coli. BL-21 cells transformed with a recombinant plasmid pEasy-H protein 1-204aa were subjected to SDS-PAGE analysis. Lane M: PageRuler^{TM} Prestained Protein Ladder (Thermo Scientific,26616); lane 1, expression of plasmid pEasy with IPTG induction 1 mM IPTG for 6 h; lane 2,expression of plasmid pEasy without IPTG induction; lane 3, expression of His-tagged H protein 1-204aa with IPTG induction 1 mM IPTG for 6 h. The objective protein shown in the red box ; lane 4, expression of His-tagged H protein 1-204aa without IPTG induction.

TABLE 1 GenBank accession numbers and genotypes of all isolates used in this study.

Accession number	Isolate	Genotype
KC494688.1	CDV SD-12-3	Asia-1
KP765763.1	CDV SD(14)7	Asia-1
KJ994343.1	CDV ZC	Asia-1
KJ848781.1	CDV RD-JL	Asia-1
HM448830.1	CDV HeB(09)1	Asia-1
JN896331.1	CDV PS	Asia-1
HQ540292.1	CDV HLJ2-07	Asia-1
KJ466106.1	CDV SY	Asia-1
JX681125.1	CDV HLJ1-06	Asia-1
EF445053.1	CDV NM	Asia-1
EU098103.1	CDV BR2	Europe
KX434626.1	CDV LDM-BTU-2	Europe
KF640687.1	CDV R252	America-2
AF164967.1	CDV A75/17	America-2
Z47759.1	CDV Denmark2004	Europe wildlife
X84999.1	German ferret isolate	Europe wildlife
AB476402.1	CDV 50Con	Asia-2
AB474397.1	CDV 007Lm	Asia-2
KC966929.1	CDV Wb-2013	Arctic
KM115535.1	CDV BA376/13 ITA	Arctic
JN896987.1	Snyder Hill	America-1
EU726268.1	CDV3	America-1
AF014953.1	Onderstpoort	America-1
Z35493.1	Convac vaccine strain	America-1
